# Applying current European periodontitis clinical practice guidelines is not feasible even for the richest countries in the world

**DOI:** 10.1111/cdoe.13003

**Published:** 2024-08-15

**Authors:** Eero Raittio, Jostein Grytten, Rodrigo Lopez, Carl Christian Blich, Mario Vianna Vettore, Vibeke Baelum

**Affiliations:** ^1^ Department of Dentistry and Oral Health Aarhus University Aarhus Denmark; ^2^ Institute of Dentistry, University of Eastern Finland Kuopio Finland; ^3^ Section of Community Dentistry University of Oslo Oslo Norway; ^4^ Center for Translational Oral Research—Periodontology, Department of Clinical Dentistry University of Bergen Bergen Norway; ^5^ School of Dentistry, Faculty of Medicine Pontificia Universidad Católica de Chile Santiago Chile

**Keywords:** clinical practice guidelines, dental care, epidemiological monitoring, health care economics and organizations, overdiagnosis, periodontitis

## Abstract

Clinical practice guidelines aim to enhance the quality, equality and consistency of care but often demand more time than is available, rendering adherence impractical and exceeding feasible resources. The 2017 introduction of a new periodontal classification system by the American Academy of Periodontology (AAP) and the European Federation of Periodontology (EFP) sought to refine clinical and epidemiological practices by serving as the basis for clinical practice guidelines and epidemiological investigations around the world. Following this classification, the EFP recommends supportive periodontal care visits every 3–12 months for all periodontitis cases. Given that in Norway, approximately 72% of the adult population are identified as periodontitis cases under the current AAP/EFP case definition, this poses a significant demand on healthcare resources. We calculated that between 60% and 70% of all estimated available working hours available for adult dental care provided by dentists and dental hygienists in Norway in 2017 would be spent on supportive periodontal care visits alone if the recommendations were to be met. This situation calls for a reevaluation of disease definitions and clinical practice guidelines to ensure they are practical, financially feasible and patient‐outcome relevant.

Clinical practice guidelines aim to improve the quality, equality and consistency of care. Even though they may advise against some ineffective and costly interventions, following the recommendations is frequently impossible due to more time being needed to follow them than is feasibly available.^1^ As an example, it has been estimated that the implementation of the 2007 European Guidelines for the Management of Arterial Hypertension in the adult population in a Norwegian county would require more time for treatment than the entire existing capacity of general practitioners could muster even if devoted to this task alone.^2^

In dentistry, prior studies have indicated that when defining population treatment needs for specific dental diseases (such as dental caries or periodontal disease) using standard clinical approaches, this results in workforce requirements that greatly exceed what is feasibly affordable, particularly for developing nations.^3^, ^4^, ^5^ However, we are not aware of recent investigations of the feasibility of current clinical practice guidelines in affluent Western countries.

In 2017, the World Workshop on the Classification of Periodontal and Peri‐implant Diseases and Conditions organized by the American Academy of Periodontology (AAP) and European Federation of Periodontology (EFP) resulted in the development of a new periodontal classification. Given the expanding body of literature, this new classification seems to have received a largely positive response from the dental community, and it has been introduced as the basis for clinical treatment practice guidelines by the EFP^6^, ^7^ and epidemiological investigations^8^, ^9^, ^10^ around the world.

Some of us have recently demonstrated that the new classification system did not differ from previous systems in its presuming approach to case definitions and no evidence has been presented for the superiority of the new classification system over previous models for the prediction of clinically important outcomes.^11^ Furthermore, its development was not based on a balanced assessment of the potential benefits and harms associated with its implementation. It therefore remains unknown whether the use of that system provides more net benefits to patients and to the community than previous classification systems. What is evident, however, is that the use of this classification for epidemiological purposes results in prevalence estimates^8^, ^9^, ^10^, ^12^ that do not align well with the public health importance of periodontitis, and overdiagnosis is likely to be a considerable problem leading to an unfavourable benefits‐to‐harms ratio for major sections of any given population.

It is also problematic that the EFP periodontitis clinical practice guidelines^6^, ^7^ developed for the management of the four stages of periodontitis defined by the new classification system are not based on evidence emerging from the treatment of these stages but on evidence preceding the new classification system.^11^ None of the systematic reviews used to underpin the recommendations (available from J Clin Periodontol 2020; 47(Spec 22): 1–391, and J Clin Periodontol 2022; 49(Spec 24): 1–358) are thus specific to any of the disease categories defined in the new classification system. It therefore remains to be shown whether, or by how much, patients classified as Stage I or Stage II would receive patient‐important benefits from the treatments recommended. As previously argued,^11^ patients in these two stages ‘*are likely to have a low risk of patient‐important outcomes over their life‐course and the incremental benefits (or losses) with (or without) treatment are likely to be minor*.’ Even so, the treatment recommendation for Stage I and Stage II patients is the same as for Stage III patients, and the new classification system is therefore clearly not very helpful in guiding the selection of the better management options for the different stages of periodontitis. The management of Stage IV patients largely differs from that of Stage III patients with respect to the additional rehabilitation treatments necessary to compensate for the ‘*anatomical and functional sequelae of tooth and periodontal attachment loss (tooth flaring and drifting, bite collapse, etc.)*.’^6^

Another important aspect of the management of periodontitis at any stage is the supportive periodontal care (SPC) following active treatment. According to the current EFP periodontitis clinical practice guidelines, SPC ‘*is aimed at maintaining periodontal stability in all treated periodontitis patients*.’^7^ In SPC, preventive and therapeutic interventions are ‘*rendered at regular intervals according to the patient's needs, and in any of these recall visits, any patient may need re‐treatment if recurrent disease is detected, and in these situations, a proper diagnosis and treatment plan should be reinstituted*.’^7^ For all stable and unstable stage I–III periodontitis patients, EFP recommends that SPC ‘*visits should be scheduled at intervals of 3 to a maximum of 12 months and ought to be tailored according to patient's risk profile and periodontal conditions after active therapy.*’^7^ In stage IV periodontitis patients EFP recommends that SPC ‘*should initially be provided at 3‐monthly intervals. Medium to long‐term frequency should be personalized to each individual patient, taking into account clinical and behavioural circumstances*.’^6^ According to the guideline, SPC visits usually require 45–60‐min appointments.^7^ Again, these recommendations have not been derived from evidence pertaining to each of the periodontitis stages concerned, and their necessity and relevance remains uncertain.

What is easily demonstrable, however, is that the above SPC recommendations are quite out of proportion to the available resources regardless of country. For this study, we have estimated the clinical time needed to implement the EFP clinical practice recommendations regarding SPC in one Norwegian county (Nord‐Trøndelag) in 2017. In Nord‐Trøndelag, a population‐based clinical study on periodontitis prevalence was conducted between 2017 and 2019.^10^ The study included clinical and radiographical periodontal data for almost 5000 participants aged over 19 years as part of the fourth Trøndelag health survey (HUNT4). By applying the 2017 AAP/EFP periodontitis classification, the total periodontitis prevalence was estimated at 72.4%. The Stage I periodontitis prevalence was 13.8%, Stage II 41.1%, Stage III 15.3% and Stage IV 2.3%.^10^ These prevalence estimates are in line with those reported from studies conducted elsewhere using the 2017 classification criteria.^8^, ^9^

In Norway, adults receive dental treatment from private practitioners, and the costs are primarily covered by patient fees, which are freely set by market forces. However, treatment of periodontitis, including the prosthetic rehabilitation after periodontitis treatment, receives a substantial public subsidy.^13^ The number of dentists per capita in Norway surpasses that of most other countries, and the dental workforce is evenly distributed across regions.^14^, ^15^ Statistics related to dental workforce and the population are accessible via Statistics Norway^14^, ^16^ and these were used to assess whether the estimated needs could be met.

Table 1 shows the results of the estimation of the clinical time needed to follow the SPC recommendations outlined in the EFP clinical practice guidelines for the management of periodontitis.^6^, ^7^ If the EFP recommendations were to be met, 71% of all estimated available clinical hours available for adult dental care provided by dentists and dental hygienists in the county of Nord‐Trøndelag in 2017 would be spent on SPC visits alone. When we carry out the calculations for whole Norway, 61% of available clinical hours available for adult dental care would be spent on SPC visits alone. It is worth noting that we did not consider the time needed to conduct the examinations and treatments (the steps 1–3 in the EFP guideline jargon) that invariably precede any SPC: periodontal examinations/diagnostics, active treatments, re‐treatments, re‐evaluations of periodontitis and surgical periodontal procedures. Nor did we consider any treatment that might be necessary for other dental conditions, although we recognize that SPC visits could offer some benefits in managing other dental conditions as well. Not only do these estimates therefore indicate that the EFP clinical practice guidelines for the management of periodontitis are clearly out of proportion to the resources available, but they also highlight the rather arbitrary nature of the recommendations which seem founded in traditions rather than in careful consideration of the real needs let alone the consequences for clinical practice, patients or society.

**TABLE 1:** Estimation of time needed for supportive periodontal care (SPC) visits in the county of Nord‐Trøndelag and in Norway. Population of adults 20 years and older.

	Nord‐Trøndelag 2017			Norway 2017			Norway 2023			References
	Stage I–III periodontitis	Stage IV periodontitis	Total	Stage I–III periodontitis	Stage IV periodontitis	Total	Stage I–III periodontitis	Stage IV periodontitis	Total	
Population size	–	–	103 366	–	–	3 995 587	–	–	4 247 557	16
Prevalence (%)	70.1	2.3	72.4	70.1	2.3	72.4	70.1	2.3	72.4	10
Estimated no. of cases	72 460	2377	74 837	2 800 906	91 899	2 892 805	2 977 537	97 694	3 075 231	
Avg. SPC visits per year	1.5	3		1.5	3		1.5	3		6,7
Avg. SPC visit duration (h)	0.75	1		0.75	1		0.75	1		7
Total hours needed for SPC visits per year (h)	81 517	7132	88 649	3 151 020	275 696	3 426 715	3 349 730	293 081	3 642 811	
No. of man‐labour years in private sector^1^			76.6			3483.6			3727.9	14
Man‐labour year in hours (h)			1620			1620			1620	17
No. working hours per year (h)			124 092			5 643 432			6 039 198	
Pct. of working hours needed for SPC visits			71.4			60.7			60.3	

*Note*: 1 = Includes general dentists, specialists in periodontics and dental hygienists.

These estimates are also out of proportion to the time allocated to periodontitis treatment in Norway. Fardal et al.^17^ analysed the national profile for the provision of treatment for periodontitis in the year 2013 and found that the ‘*prevalence of diagnosed and treated periodontitis in Norway*’ was low (estimated at 4.4% of the population) and that the frequency of periodontal treatments was ‘*sufficient to maintain major tooth retention for the population*’. This occurred at the expense of only 4% of the man‐labour years available for adult dental services being used for periodontal examinations and treatment, which is much less than what adopting the EFP SPC recommendation would require. Only about 10% of the adult Norwegian population fell in the categories of irregular dental visitors or non‐attenders,^18^ and even if it is assumed that these persons would present double the periodontitis treatment need this would not increase man‐labour years needed for periodontitis considerably.^17^ It seems that providing SPC visits at the scale recommended by the EFP has not resulted in, nor is it necessary for maintaining high tooth retention for the population.

In line with all other epidemiological studies using the criteria of the new AAP/EFP periodontitis classification system, the studies emerging from Norway have indicated exorbitantly large periodontitis estimates amongst Norwegians.^10^, ^12^, ^19^ This may be a one explanation for the considerable increase in the public subsidy for the cause‐related treatment of periodontitis from 21.1 million Euros in 2013 (before the introduction of the new classification system) to 53.6 million Euros in 2022 (after the introduction), representing an increase of 83% (Figure 1). These figures are deflated by the consumer price index for dental services. The increase without adjusting for inflation is 154%.

**FIGURE 1:**
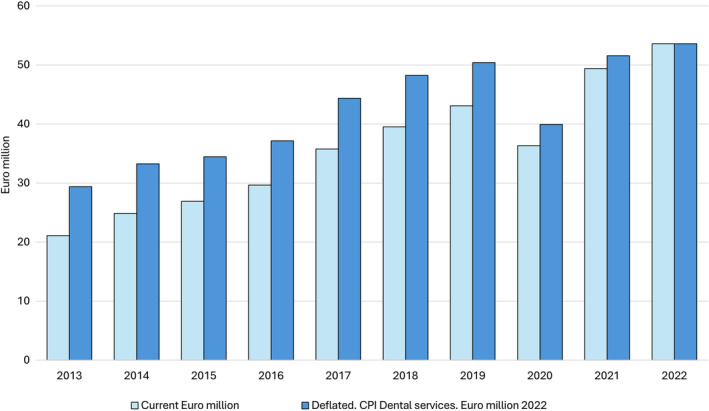
Reimbursements (in million Euros) for treatment of periodontitis in Norway 2013–2022. CPI, consumer price index. Norwegian Directorate of Health. The KUHR database. https://www.helsedirektoratet.no/tema/statistikk‐registre‐og‐rapporter/helsedata‐og‐helseregistre/kuhr.

This raises the rather pertinent question of whether this increment reflects a recent outbreak of periodontitis amongst Norwegians, or whether the apparent epidemic has been created by the dental profession itself through the implementation of the new classification system.^20^ Indications that the latter is the case is found in the observation that implausibly high disease prevalence and workforce requirement estimates accompanied by low actual spending on periodontal care are not new phenomena and has occurred with earlier approaches for estimating periodontal treatment needs, the Community Periodontal Index of Treatment Needs and Russell's Periodontal Index.^3^, ^5^, ^21^, ^22^, ^23^

In our view, these Norwegian data support the criticisms raised against the 2017 AAP/EFP periodontitis classification system in that it is likely to lead to overdiagnosis and overtreatment and will shift the benefits‐to‐harms ratio for periodontitis treatment in a negative direction for both patients and for society at large.^11^ Aligning with the perspective presented by Johansson et al.,^1^ the only practical way forward involves a reevaluation of disease definitions (i.e. a revision of the new periodontitis classification system) and the treatment thresholds, duration and frequency (i.e. the clinical treatment practice guidelines for periodontitis), aiming to attain levels that are both practically and financially feasible and relevant for patient‐important outcomes. In the UK, an alternative approach is taken and the EFP guidelines are now implemented so that more resources are used on ‘*engaging patients*’ who represent adequate oral hygiene and risk factor control in reevaluation, whereas the ‘*non‐engaging patients*’ are recommended to receive palliative periodontal care.^24^ In our view, people and societies who apply and promote such approaches have the burden of proof whether this approach results in better patient‐important outcomes than alternative, less invasive and resource‐demanding approaches for the whole population, irrespective of engagement.

It would of course require a separate deliberate multidisciplinary process to propose proper alternative periodontal disease definitions and clinical practice guidelines. Fortunately, there is a considerable amount of guidance and insights^1^, ^25^, ^26^, ^27^, ^28^, ^29^, ^30^, ^31^ that the periodontal community could consider when revising disease definitions and clinical practice guidelines to alleviate the evident overdiagnosis and overtreatment burden. To support this shift further, there is a critical need for robust evidence demonstrating the relationship of periodontal measurements with patient‐important outcomes, as well as demonstrating the effectiveness of treatments, such as SPC, in improving patient‐important outcomes.^32^, ^33^

## FUNDING INFORMATION

This research received no external funding.

## CONFLICT OF INTEREST STATEMENT

The authors declare that they have no known competing financial interests or personal relationships that could have appeared to influence the work reported in this paper.

## PATIENT CONSENT STATEMENT

Not required for this type of article.

## PERMISSION TO REPRODUCE MATERIAL FROM OTHER SOURCES

Not required for this type of article.

## CLINICAL TRIAL REGISTRATION

Not required for this type of article.

## Data Availability

The authors confirm that the data supporting the findings of this study are available within the article or its supplementary materials.
